# Perioperative Dexmedetomidine Fails to Improve Postoperative Analgesic Consumption and Postoperative Recovery in Patients Undergoing Lateral Thoracotomy for Thoracic Esophageal Cancer: A Randomized, Double-Blind, Placebo-Controlled Trial

**DOI:** 10.1155/2020/4145893

**Published:** 2020-05-06

**Authors:** Yu Mao, Xuemei Sun, Li Si, Lijian Chen, Xuesheng Liu, Zhi Zhang, Erwei Gu

**Affiliations:** ^1^Department of Anesthesiology, First Affiliated Hospital of Anhui Medical University, Jixi Road 218, Hefei City, Anhui Province, China; ^2^School of Life Sciences, University of Science and Technology of China, Huangshan Road 443, Hefei City, Anhui Province, China; ^3^Department of Laboratory, First Affiliated Hospital of Anhui Medical University, Jixi Road 218, Hefei City, Anhui Province, China

## Abstract

**Objectives:**

Dexmedetomidine is widely used as an adjunct to general anesthesia. In this study, we evaluated the effects of perioperative dexmedetomidine infusion on postoperative analgesia in patients undergoing lateral thoracotomy for thoracic esophageal cancer.

**Methods:**

A total of 62 patients undergoing lateral thoracotomy for thoracic esophageal cancer were randomized to receive adjuvant therapy with either dexmedetomidine (0.5 *μ*g/kg intravenous bolus injection for 10 min before induction of anesthesia, followed by continuous infusion of 0.2–0.4 *μ*g/kg/h until the end of surgery, and 0.06 *μ*g/kg/h for 5 days after surgery) or equal volumes of saline. Acute postoperative pain was treated with patient-controlled intravenous sufentanil and flurbiprofen axetil. The primary outcomes of this study were the numbers of analgesic requirements in the first postoperative 72 h.

**Results:**

Perioperative dexmedetomidine did not decrease the numbers of analgesic requirements in the first postoperative 72 h (dexmedetomidine group: 12.14 ± 4.76, saline group: 10.89 ± 5.66; *p*=0.367). Likewise, the groups did not differ with respect to total postoperative analgesic requirements, postoperative pain, perioperative inflammation, blood cell count, incidence of adverse events, surgical recovery (assessed at postoperative days 2 and 5 using the surgical recovery scale), length of hospital stay, hospital cost, incidence of chronic pain, or quality of life. Notably, dexmedetomidine had beneficial effects on decreasing intraoperative opioid consumption and improving postoperative sleep quality. *Discussion*. Perioperative dexmedetomidine has limited analgesic benefits in lateral thoracotomy for esophageal cancer when added to an opioid-based multimodal anesthetic regimen but can reduce opioid consumption.

## 1. Introduction

Postoperative pain management after thoracic surgery remains a challenge [1–4]. The underlying mechanisms of acute postoperative pain in thoracotomy are likely multifactorial and are mainly due to direct surgical trauma to ribs, costovertebral joints, intercostal nerves, and/or pleural irritation from chest tubes [3]. Adequate postoperative analgesia is imperative for reducing morbidity in postthoracotomy patients, improving functional outcomes, enhancing short-time recovery, and preventing the chronification of acute pain [5–7].

Opioids are still considered mainstream for postoperative pain control but cause adverse effects including sedation, pulmonary depression, constipation, urinary retention, pruritus, hyperalgesia, postoperative nausea, and vomiting [8, 9]. These adverse effects are not only associated with perioperative morbidity and mortality but can also delay hospital discharge and increase hospital costs. Therefore, modern analgesic strategies aiming at reducing opioid-induced side effects represent a multimodal approach [2, 4, 10].

Dexmedetomidine is a highly selective *α*2-adrenergic receptor agonist that has sedative and analgesic-sparing effects [11, 12], perioperative sympatholysis [13], and reduced postoperative delirium and agitation [14–16]. In patients undergoing lateral thoracotomy for pulmonary resection, perioperative administration of dexmedetomidine for postoperative pain control decreases opioid consumption, provides effective postoperative analgesia, and improves patient satisfaction. However, data on the efficacy and safety of dexmedetomidine in lateral thoracotomy for thoracic esophageal carcinoma are lacking [17, 18].

We hypothesized that, in patients undergoing lateral thoracotomy for thoracic esophageal cancer, perioperative dexmedetomidine infusion would reduce opioid requirements and provide postoperative pain relief for patients who are not available for neural blockade. In the present study, we assessed peripheral cell counts, the pattern of inflammatory responses, sleep quality, surgical recovery, the incidence of chronic pain, and quality of life after surgery.

## 2. Methods

### 2.1. Study Design and Population

A total of 62 patients undergoing lateral thoracotomy for thoracic esophageal cancer were enrolled in this prospective, double-blinded randomized, placebo-controlled trial.

The study protocol was approved by the Human Research Ethics Committee of Anhui Medical University (20180224) and was registered in the Chinese Clinical Trial Register (No. ChiCTR 1800015054, Principal Investigator: Yu Mao, Date of Registration: March 5, 2018). Patients were enrolled between March and July of 2018. Inclusion criteria consisted of age being 18–75 years and an American Society of Anesthesiologists (ASA) physical status of I–III. The exclusion criteria included the following: hypersensitivity to dexmedetomidine, conduction abnormalities other than atrioventricular block, bradycardia, rate-controlled atrial fibrillation, abnormal ventricular ejection (defined as left ventricular ejection fraction <50%), liver impairment (defined as total serum bilirubin ≥34.2 *μ*M), renal disease (defined as glomerular filtration rate ≤60 mL/min/1.73 m^2^), serious central nervous system pathology, chronic pain, psychiatric disorder, epilepsy, communication disability, or psychological/physical dependence on opioids (e.g., transdermal fentanyl or morphine). Informed consent was obtained from patients prior to initiation of the study. Demographic and other confounding variables including age, gender, ASA status, height, weight, surgical recovery scale, and sleep quality were collected in ward the day before surgery. Patients were randomized to either a saline or dexmedetomidine group using a computer-generated random sequence of numbers with a 1 : 1 allocation ratio. Allocation concealment was achieved by concealing assignments in sequentially numbered, opaque, sealed envelopes, which were opened after the arrival of the patient in the operating room. Blinding of research personnel and the staff involved in the study was maintained throughout the study and during all of the postoperative follow-ups.

### 2.2. Study Intervention

Patients in the dexmedetomidine group were intravenously infused with dexmedetomidine at a loading dose of 0.5 *μ*g/kg for 10 min before induction of anesthesia, followed by continuous infusion of 0.2–0.4 *μ*g/kg/h until the end of surgery, which was continued at 0.06 *μ*g/kg/h for 5 days after surgery [12, 17–19]. Patients in the saline group were administered equivalent volumes of saline via the identical scheme as that for the dexmedetomidine group. The study drugs were prepared by an anesthetist, who was not a member of the research team and did not participate in administering the anesthesia or follow-up of the study participants. The study medication was prepared in a 20 mL syringe (for continuous infusion during anesthesia) and a 250 mL pump (for the postoperative continuous infusion at 2 mL/h). The 20 mL syringe contained 10 *μ*g/mL of dexmedetomidine solution or an equal volume of 0.9% saline. The 250 mL pump contained either 7.2 mg/kg of dexmedetomidine (0.06 *μ*g/kg/h) or a 0.9% saline solution.

### 2.3. Anesthesia Protocol

General anesthesia was induced with midazolam (0.05 mg/kg), sufentanil (0.5 *μ*g/kg), and a target-controlled infusion (TCI) of propofol using the Marsh model [20, 21] (Graseby 3500 Anesthesia Pump; Graseby Medical Ltd., Lower Pemberton, UK). The initial target effective concentration was 1.0 *μ*g/mL, which gradually increased by 0.3 *μ*g/mL until the bispectral index (BIS; Vista; Aspect Medical Systems Inc., Norwood, Norfolk County, MA, USA) value was below 60. Rocuronium (0.9 mg/kg) was administered for double-lumen endotracheal tube placement. Anesthesia was maintained with continuous TCI of propofol and remifentanil after intubation, and one-lung ventilation was applied during surgery. The rate of propofol was titrated to maintain a BIS value between 40 and 60, and the doses of intraoperative remifentanil, rocuronium, and sufentanil were titrated according to the discretion of the anesthetist. After completion of the surgical procedure, patients were admitted to the postanesthesia care unit (PACU) and were extubated. An injection of flurbiprofen axetil (1 mg/kg) was provided prior to surgery, whereas sufentanil (0.1–0.2 *μ*g/kg) and flurbiprofen axetil (1 mg/kg) were administered at the end of surgery, followed by patient-controlled intravenous (i.v.) analgesia (PCIA) of a mixture of sufentanil and flurbiprofen axetil in 250 mL at a concentration of 0.03 *μ*g/kg/mL and 1 mg/mL, respectively. The PCIA pump included a background infusion of 2 mL/h without a loading dose and a demand dose of 2 mL/h with a lockout interval of 15 min. An additional bolus of PCIA was given by the nurses in the PACU if the visual analog scale (VAS) for pain exceeded 3. Prophylaxis against postoperative nausea and vomiting (PONV) was performed with dexamethasone (0.2 mg/kg [max, 8 mg]) before the induction of anesthesia and azasetron (0.2 mg/kg [max, 10 mg]) at the end of surgery. Pantoprazole (40 mg, i.v.) was administered to treat PONV if the VAS for nausea and vomiting exceeded 3.

### 2.4. Study Outcomes

The primary outcome was the effective pressing numbers of PCIA-boli during the first postoperative 72 h. Secondary endpoints included the following: (1) severity of postoperative pain at rest and movement, as evaluated with the VAS (0 = no pain, 10 = worst imaginable pain) once daily on postoperative days (PODs) 1, 2, and 5; (2) incidence of PONV during the first 5 PODs; (3) sleep quality, as assessed using St. Mary's Hospital Sleep Questionnaire [22], once daily on PODs 2 and 5 including sleep quality (1 = worst possible sleep, 6 = best possible sleep), being clear-headed after getting up in the morning (1 = most drowsy, 6 = best alertness), and sleep satisfaction (1 = most unsatisfied, 5 = most satisfied); (4) inflammatory markers, as examined by the serum concentrations of interleukin-6 (IL-6), IL-10, and C-reactive protein (CRP) and measured at four distinct time points: T0, prior to induction of anesthesia (baseline); T1, at the end of the operation; T2, the morning of the second POD; and T3, the morning of POD 5; (5) full blood count, serum glucose, and immune cell subset analysis, as measured at three time points: T0, prior to induction of anesthesia (baseline); T1, the morning of POD 2; and T2, the morning of POD 5; (6) short-term recovery (defined as the time to awakening, extubation, first movement of leaving the bed, first intestinal aerofluxus, hospitalization cost, length of PACU stay, hospital stay, and postoperative hospital stay); (7) surgical recovery scale (SRS) [23], as evaluated 1 day prior to surgery and on PODs 2 and 5 (each item response was scored in order from “1” to “5” or “6,” depending on the number of possible responses for each item); (8) incidence of chronic postsurgical pain at 3 months after surgery; and (9) quality of life as measured with the Short Form 8 (SF-8) health survey [24] (each item response was scored in order from “1” to “5” or “6,” depending on the number of possible responses for each item) at 1 day prior to surgery and at 1 and 3 months after surgery (administered by phone) [25].

### 2.5. Safety

The study drug was administered to patients with close hemodynamic monitoring in a completely equipped operative room and ward. This facilitated immediate detection and management of adverse events including bradycardia, tachycardia, hypotension, and hypertension [11, 16]. Bradycardia was defined as a heart rate < 55 bpm or *a* > 20% decrease from baseline (in the case of a prestudy drug infusion value < 69 bpm). Tachycardia was defined as heart rate >100 bpm or *a* > 20% increase from baseline (in the case of a prestudy drug infusion value > 83 bpm). Hypotension was characterized as mean blood pressure <60 mm Hg or *a* > 20% decrease from baseline (in the case of a prestudy drug infusion value < 75 mmHg). Hypertension was characterized as systolic blood pressure >160 bpm or *a* > 20% increase from baseline (in the case of a prestudy drug infusion value > 133 mmHg). Administration of study medication was immediately adjusted or stopped when participants showed dexmedetomidine-associated adverse reactions.

### 2.6. Blood Sample Acquisition and Laboratory Methods

In patients from both groups, 4 mL of venous blood samples in EDTA was obtained before the induction of anesthesia (T0), at the end of surgery (T1), on POD 2 (T2), and on POD 5 (T3). A full blood count of a 2 mL blood aliquot from three time points (T0, T2, and T3) was analyzed using the hematology analyzer, Sysmex XE-2100 (Sysmex, Kobe, Japan). A volume of 2 mL of blood samples from four time points (T0, T1, T2, and T3) was immediately centrifuged at 2,500 rpm for 15 min and was then stored at −80°C until subsequent analysis of IL-6, IL-10, and CRP concentrations. Inflammatory markers were measured using commercial enzyme-linked immunosorbent assay (ELISA) kits (Human CRP/IL-6/IL-10 Quantikine ELISA Kits; R&D Systems, Inc., Minneapolis, MN, USA). CRP was expressed as *μ*g/mL, whereas IL-6 and IL-10 were expressed as pg/mL. The coefficient of variation of the assay for the entire sample batch was 10.8%.

### 2.7. Statistical Analysis

The sample size calculation, based on our pilot data and previous study [26] of differences in effective pressing numbers of PCIA during the first postoperative 72 h, indicated that a total of 55 patients were needed to provide a power of 0.8 to detect a clinically significant difference between a group mean effective pressing numbers of 12 with a pooled standard deviation (SD) of 5.6 at a level of 0.05; this equates to a 33.3% difference between means. Thus, a total of 62 patients (31 in each group) were recruited to account for potential drop-outs. All statistical analyses were performed using SPSS version 22 for Windows (SPSS Inc., Chicago, IL, USA) and GraphPad Prism version 5 (GraphPad Software, Inc., La Jolla, CA, USA). Additionally, a per-protocol analysis, excluding patients lost to follow-up, was performed. Categorical data were analyzed using the *χ* [2] test, and comparison of proportions was analyzed using Fisher's exact test. A Mann–Whitney *U* test was performed to analyze the pressing numbers of PCIA (primary outcome) and other quantitative parameters between the two groups. *p* values <0.05 were considered statistically significant.

## 3. Results

**Figure 1:**
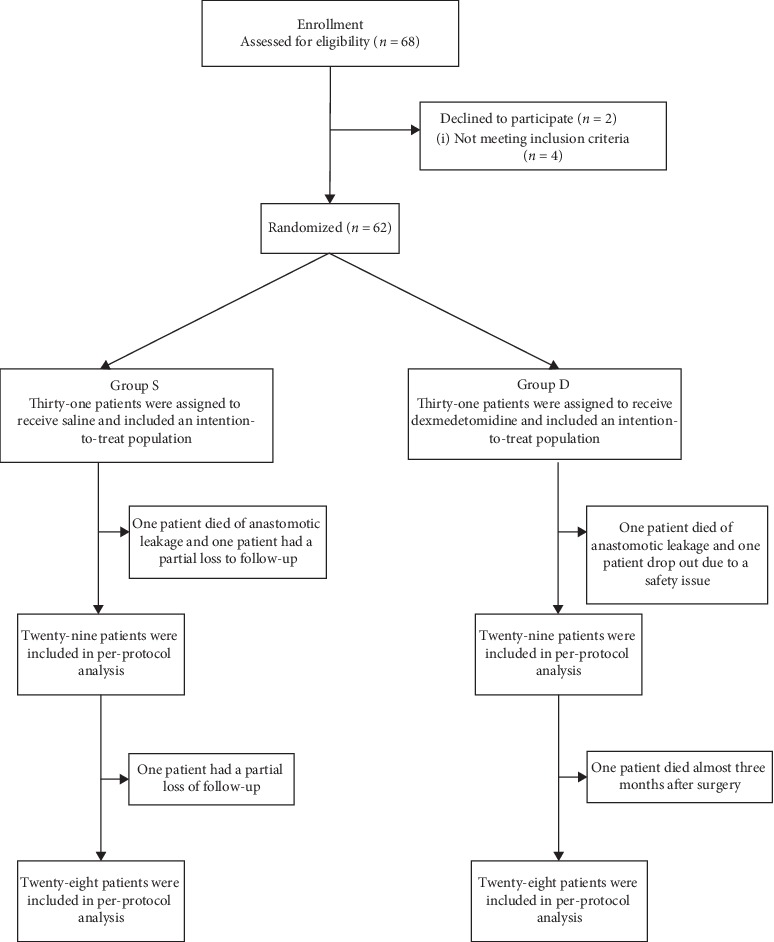
Flowchart.

The modified Consolidated Standards of Reporting Trials diagram represents the recruited patients (Figure 1) [27, 28]. A total of 68 patients were screened for lateral thoracotomy, among which 62 patients were randomized to treatment with dexmedetomidine (*n* = 31) or saline (*n* = 31). The in-hospital per-protocol population included 58 patients, as one patient in the saline group or dexmedetomidine group died of anastomotic leakage, one patient in the saline group was lost to follow-up, and one patient in the dexmedetomidine group was excluded due to hemodynamic instability. The per-protocol population at 3 months after surgery was 56 patients because one patient in the saline group was lost to follow-up and one patient in the dexmedetomidine group died at almost 3 months after surgery, precluding any assessment of quality of life.

### 3.1. Preoperative Assessment and Intraoperative Data

There was no difference between the two groups with respect to patient characteristics (Table 1). Surgery, anesthesia, and PACU-related data were comparable between the two groups, with the exception of sufentanil and remifentanil consumption, which was significantly higher in the saline group than in the dexmedetomidine group (*p*=0.008 and *p*=0.015, respectively; Table 2).

### 3.2. Primary Outcomes

Effective pressing numbers of PCIA-boli during the first postoperative 72 h were not significantly different between the saline and dexmedetomidine groups (12.14 ± 4.76 *versus* 10.89 ± 5.66, *p*=0.367; Table 2).

### 3.3. Secondary Outcomes

The two groups did not differ with respect to the VAS for pain at rest or movement at any time point (Table 2). The two groups also did not differ regarding the total numbers of PCIA-boli delivered by a pump during the first five PODs (Table 2). Sleep quality on PODs 2 and 5 and a clear-headed feeling after waking up on POD 5 were better in the dexmedetomidine group than in the saline group. Sleep satisfaction on PODs 2 and 5, the PONV incidence during the first five PODs, time to the first out-of-bed activity, time to removal of the chest drainage tube, time to the first intestinal aerofluxus, time until discharge, time of postoperative stay, and hospital cost were not significantly different between the two groups (Table 2). Likewise, the SRS survey did not differ between the two groups on PODs 2 and 5 (Table 2). In addition, the SF-8 generic health survey was comparable between the two groups at both 1 and 3 months after surgery, and the incidence of chronic postsurgical pain did not differ between the two groups (Table 3).

### 3.4. Laboratory Findings

Red blood cells (RBCs), hemoglobin, platelets, and white blood cells (WBCs)—including basophils, eosinophils, and monocytes—did not significantly differ between the two groups on PODs 2 and 5 (Table 4). Meanwhile, the serum concentrations of CRP, IL-6, and IL-10 were comparable between the two groups at all time points (Table4).

### 3.5. Postoperative Safety Data

There were no significant differences in the incidence of adverse events between groups (Table 2). Bradycardia was noted in one patient (who was in the dexmedetomidine group) after pressing one bolus of PCIA on the operative day, and this issue was resolved after the infusions of PCIA and dexmedetomidine were stopped. No hemodynamic instability was observed after adjusting postoperative analgesia to half the concentration of PCIA and dexmedetomidine. Ultimately, this patient was excluded from this trial.

## 4. Discussion

Our present study represents the first in-depth and systematic evaluation of the effects of perioperative use (i.v. dexmedetomidine) on postoperative analgesia, postoperative recovery, and cellular/inflammatory components of the immune system after surgery. However, dexmedetomidine failed to decrease postoperative opioid consumption, relieve postoperative pain, attenuate inflammation, lessen changes in blood cell counts, reduce the incidence of postoperative complications, accelerate postoperative short-term recovery, decrease the incidence of chronic postsurgical pain, or improve quality of life after discharge.

Our present results contradict the observations of three published studies that also investigated the efficacy of systemic dexmedetomidine in thoracotomy surgery [17, 18, 29]. These trials reported significant effects of postoperative i.v. dexmedetomidine on pain intensity [18, 29] and a reduction in opioid consumption [17, 18, 29]. Several nonthoracotomy studies—including hysterectomy [19, 30, 31], inpatient surgery [32], spinal surgery [33], neurosurgery [34], radical mastectomy [35], and open gastrectomy [36]—have also demonstrated significant analgesic effects of dexmedetomidine. The findings of our present study are also in contrast with several meta-analyses that exhibited significant analgesic and opioid-sparing effects for the perioperative administration of systemic dexmedetomidine [12, 37–39]. Of note, these previous meta-analyses included patients undergoing different surgical procedures, so these data on the analgesic efficacies of dexmedetomidine are not directly comparable to the results of our present study. Nevertheless, it may be worthwhile to speculate why our present results, in contrast to those of the above-mentioned trials and meta-analyses, demonstrated that dexmedetomidine had no significant postoperative analgesic effects.

First, the patients underwent lung thoracotomies in one trial [17, 18], whereas those undergoing esophageal thoracotomy in the other trial were highly nicotine-dependent patients [29]. Besides that, patients underwent different types of thoracotomy including esophageal neoplasia resection, lobectomy, pneumonectomy, mediastinal mass resection, and pneumothorax [18]. The subjects considerably differed from our present study. All of the patients in our present study underwent complex and extensive surgery for radical esophageal carcinoma via a single incision. Second, the dose with which systemic opioid was infused in our research was higher than that in the three other trials investigating the efficacy of dexmedetomidine in thoracotomy [17, 18, 29]. In our present study, a remifentanil infusion was started after induction of anesthesia and continued until the completion of surgery, whereas remifentanil was not administered in these previous studies. In addition, paravertebral regional block was provided in one previous trial [17], while our patients just received 0.1–0.2 *μ*g/kg sufentanil at the end of thoracotomies without neural blockade. Moreover, PCIA with continuously infused sufentanil (0.06 *μ*g/kg/h) was higher than that in the other three trials; analgesic effects of dexmedetomidine may have been masked by the perioperative use of opioids. Third, flurbiprofen axetil, a nonsteroidal anti-inflammatory drug perioperatively administrated in our present study, plays an essential part of analgesia in multimodal analgesia [40–42]. Likewise, postoperative analgesic effects of dexmedetomidine may be hidden when flurbiprofen axetil is infused perioperatively. Fourth, the dose with which postoperative i.v. dexmedetomidine was infused in our trial (0.06 *μ*g/kg/h) was lower than that in one trial researching the efficacy of dexmedetomidine titrated from 0.1 to 0.5 *μ*g/kg/h after surgery for up to 24 h in thoracotomy [17]. In our present study, a bolus was given before induction of anesthesia, and the infusion of dexmedetomidine was continued until POD 5. This likely resulted in a higher cumulative dexmedetomidine dose when compared with that of a previous study [17]. Moreover, our dose of the dexmedetomidine infusion was higher than that used in the other two studies [18, 29]. Therefore, the failure of dexmedetomidine to reduce postoperative opioid requirements cannot be attributed to underdosing.

It has been reported that intraoperative dexmedetomidine administration is beneficial for the recovery of gastrointestinal motility function after resection of colorectal cancer, with stable hemodynamics during surgery [43]. In lower gastrointestinal surgery, postoperative ileus is mainly attributed to both inflammation and direct injury to gastrointestinal tissue. Dexmedetomidine counteracts both of these mechanisms by protection against intestinal epithelial barrier disruption [44] and inhibiting the inflammatory response [45]. In contrast, thoracotomy for esophageal cancer does not trigger postoperative ileus by direct injury to the intestines or manipulation/distraction of the plexuses of splanchnic nerves, which are located anterior to the lumbar column [40]. Consequently, we did not observe a significant reduction in the time to recovery of intestinal function in our present study.

Cytokines and immune cells are crucially important in several mechanisms underlying pain [46, 47] and postoperative recovery [48, 49]. Proinflammatory cytokines and anti-inflammatory cytokines are involved in modulating pain sensitivity and, conversely, can also be influenced in synthesis and release during pain stimuli [50]. Dexmedetomidine has anti-inflammatory effects by attenuating the plasma levels of proinflammatory cytokines [51–53]. Thoracotomy for esophageal cancer is not only associated with severe postoperative pain but also provokes a tremendous systemic inflammatory response [54]. The inflammatory reaction significantly affects postoperative recovery, as excessive stimulation of the inflammatory cascade can predict the outcome after surgery and lead to infection, pain, systemic inflammatory response syndrome, and organ dysfunction [47, 55]. However, dexmedetomidine failed to exert anti-inflammatory or analgesic effects in our patients.

Our findings indicate that perioperative use of dexmedetomidine reduced intraoperative opioid consumption and improved postoperative sleep quality in patients undergoing lateral thoracotomy for thoracic esophageal cancer. These results are in accordance with the results of previous trials [56–59], in which dexmedetomidine decreased opioid consumption during intraoperative infusion and improved sleep quality during postoperative administration.

Chronic pain remains a challenge in pain control after thoracic surgery, and the incidence of chronic postsurgical pain increases when acute postoperative pain is not adequately controlled [3]. Jain et al. [60] found a benefit of dexmedetomidine on the incidence and severity of chronic pain at 3 months after breast cancer surgery; however, few studies have focused on chronic pain after thoracotomy with perioperative administration of dexmedetomidine. In our present study, the incidence of chronic pain (21% in the saline group and 20% in the dexmedetomidine group) remained low compared with the incidences reported in the literature [61, 62]. These results may be attributed to multimodal analgesia with opioid and flurbiprofen axetil associated with the application of PCIA.

Our present study had several limitations. First, it is debatable whether the VAS for pain is an adequate tool for evaluating the effects of dexmedetomidine on pain control. Hence, further establishment of whether VAS for pain is an adequate tool for dexmedetomidine to evaluate pain control is needed in future studies. The VAS is significantly influenced by the dosage of opioids that are administered. However, postoperative pain medication, including sufentanil and flurbiprofen axetil, was standardized in our present study and no significant difference was found in the analgesic requirements between the two groups. A second limitation of our present study is that we did not measure the dexmedetomidine concentrations in our patients. However, the dose of dexmedetomidine treatment used in our study has been shown to be effective and safe in other clinical trials [17, 18, 29]. Third, the sample size estimation in our study was based on the postoperative opioid-sparing effect of dexmedetomidine, which was powered solely as the primary endpoint in our present study. It cannot be excluded that statistically significant differences in secondary outcomes may have become apparent after the inclusion of a larger sample size. Fourth, the infusion of dexmedetomidine during anesthesia causes increasing in blood pressure and reduction of heart rate within acceptable range, which is difficult for the anesthesiologist to be blind. However, the stuff to follow-up were not the intraoperative anesthesiologists, which minimizes bias. Fifth, the practice of intraoperative administration of remifentanil for analgesia is highly debated. Remifentanil is reportedly associated with tolerance and hyperalgesia during the postoperative period [63]; however, its use was required in our present study to allow early extubation in the majority of patients.

It is noteworthy that the optimal dosing titration frequency and dosage of dexmedetomidine that balance safety and efficacy for postoperative analgesia are currently unknown. In our present study, the dosage of postoperative dexmedetomidine was 0.06 *μ*g/kg/h, although one study reported a higher dosage [18]. In our present study, only one patient experienced bradycardia, which resolved after the infusions of PCIA and dexmedetomidine were terminated. Other patients were not influenced by bradycardia, but our sample size may not have been large enough to detect such a change. Therefore, further studies are needed to determine the optimal dosage of postoperative dexmedetomidine in lateral thoracotomy for esophageal cancer with a lower incidence of hypotension and bradycardia.

## 5. Conclusions

In conclusion, the results of our present study indicate that perioperative use of systemic dexmedetomidine can decrease intraoperative opioid consumption and improve postoperative sleep quality; furthermore, this dexmedetomidine treatment had no effect on postoperative analgesia, the immune system, or short- or long-term recovery when added to opioid-based multimodal analgesia. These findings provide new evidence that adding dexmedetomidine may only provide limited benefit in thoracotomy surgery when acute pain is controlled with conventional multimodal analgesia. Further research should focus not only on the optional dosage of perioperatively administrated dexmedetomidine but also on clear identification of the effect of dexmedetomidine on the immune system and the putative mechanism of its analgesic action.

## Figures and Tables

**Table 1:** Patient characteristics.

		Saline (*n* = 29)	Dexmedetomidine (*n* = 29)	*p* value
Age	Years	63.48 ± 8.44	65.21 ± 7.31	0.409
Gender	*n* (%)			0.738
Male		24 (82.76)	23 (79.31)	
Female		5 (17.24)	6 (20.69)	
Height	Cm	166.10 ± 7.00	165.76 ± 7.32	0.855
Weight	Kg	60.45 ± 14.88	56.59 ± 11.18	0.268
BMI	kg/m^2^	21.09 ± 5.13	20.47 ± 2.95	0.196
ASA	*n* (%)			0.788
I		12 (41.38)	11 (37.93)	
II		17 (58.62)	18 (62.07)	
Surgical recovery scale		61.2 ± 11.39	62.3 ± 12.01	0.729
Sleep quality		5.00[4.00–5.00]	5.00[5.00–5.00]	0.087
Clear-headed after getting up		5.00[4.00–5.00]	5.00[4.00–5.00]	0.273
Sleep satisfaction		4.00[4.00–4.00]	4.00[3.00–4.00]	0.192

Data are presented as absolute numbers (*n*), percentages of the total population (*N*), and the mean ± standard error or median (interquartile range), as appropriate.

**Table 2:** Intraoperative and postoperative data.

			Saline (*n* = 29)	Dexmedetomidine (*n* = 29)	*p* value	Difference between mean/median	95% confidence interval
Intraoperative data	Duration of surgery	min	168.72 ± 50.57	168.62 ± 61.83	0.994	0.103 ± 14.832	[−29.609, 29.816]
	Duration of anesthesia	min	205.86 ± 56.58	207.24 ± 63.02	0.930	−1.379 ± 15.728	[−32.886, 30.127]
	Duration of one lung ventilation	min	123.21 ± 75.22	106.38 ± 71.90	0.388	16.828 ± 19.322	[−21.879, 55.534]
	Cumulative propofol dose	mg	976.92 ± 434.06	905.17 ± 397.00	0.514	71.745 ± 109.232	[−147.073, 290.563]
	Cumulative remifentanil dose	mg	2.48 ± 1.24	1.66 ± 1.25	0.015^*∗*^	0.817 ± 0.327	[0.163, 1.472]
	Cumulative sufentanil dose	*μ*g	58.52 ± 10.41	51.12 ± 9.95	0.008^*∗*^	7.397 ± 2.674	[2.040, 12.753]
	Atropine	mg	0.052 ± 0.14	0.028 ± 0.08	0.341	0.024 ± 0.03	[−0.037, 0.085]
	Phenylephrine	*μ*g	46.02 ± 95.21	52.41 ± 77.72	0.787	−6.207 ± 22.905	[−52.092, 39.678]
	Crystalloid volume	ml	1379.31 ± 416.94	1279.31 ± 352.93	0.328	100.000 ± 101.438	[−103.204, 303.204]
	Colloid volume	ml	672.41 ± 276.32	624.14 ± 247.35	0.486	48.276 ± 68.867	[−89.681, 186.233]
	Urine volume	ml	451.72 ± 264.40	465.52 ± 286.95	0.850	−13.793 ± 72.456	[−158.941, 131.354]
PACU data	Time to awakening	min	40.83 ± 31.83	37.03 ± 17.21	0.575	3.793 ± 6.72	[−9.668, 17.255]
	Time to extubation	min	39.24 ± 16.75	40.10 ± 16.83	0.846	−0.862 ± 4.409	[−9.694, 7.97]
	Time for discharge	min	79.38 ± 33.68	86.86 ± 42.78	0.462	−7.483 ± 10.11	[−27.737, 12.771]
	Cumulative sufentanil dose	*μ*g	1.12 ± 2.64	1.29 ± 3.82	0.842	−0.172 ± 0.861	[−1.898, 1.553]
Postoperative data	*VAS for pain*						
	POD1 at rest		2 [2−3]	2 [1−3]	0.066	−0.414 ± 0.22	[−0.855, 0.028]
	POD2 at rest		2 [1−2]	2 [1−2]	0.374	−0.172 ± 0.192	[−0.558, 0.213]
	POD5 at rest		1 [1–1.5]	1 [1−1]	0.372	−0.138 ± 0.153	[−0.445, 0.169]
	POD1 on movement		4.77 ± 1.30	4.79 ± 1.37	0.922	−0.345 ± 0.351	[−0.738, 0.669]
	POD2 on movement		3.79 ± 1.01	3.76 ± 1.15	0.904	0.034 ± 0.285	[−0.537, 0.606]
	POD5 on movement		3[2−4]	3 [2−4]	0.467	−0.241 ± 0.329	[−0.899, 0.417]
	*Pressing number of PCIA-boli*						
	The first 72 postoperative h		12.14 ± 4.76	10.89 ± 4.66	0. 317	−1.25 ± 1.229	[−3.728, 1.228]
	Total pressing number of PCIA-boli		15.03 ± 14.15	16.97 ± 11.63	0.572	−1.931 ± 3.401	[−8.745, 4.883]
	*Surgical recovery scale*						
	POD2		32.86 ± 2.49	32.97 ± 1.78	0.856	0.11 ± 0.568	[−1.029, 1.249]
	POD5		35.69 ± 2.95	36.00 ± 2.65	0.675	0.31 ± 0.736	[−1.165, 1.785]
	*Sleep quality*						
	POD2		4.00 [4.00–4.00]	4.50 [4.00–5.00]	0.004^*∗*^	0.621 ± 0.211	[0.198, 1.043]
	POD5		4.00 [3.00–4.00]	4.00 [4.00–5.00]	0.008^*∗*^	0.759 ± 0.265	[0.228, 1.290]
	*Clear*-*headed after getting up*						
	POD2		3.00 [3.00–4.00]	4.00 [3.00–4.50]	0.1102	0.379 ± 0.234	[−0.088, 0.847]
	POD5		3.00 [3.00–4.00]	4.00 [3.00–5.00]	0.0026^*∗*^	0.724 ± 0.230	[0.264, 1.184]
	*Sleep satisfaction*						
	POD2		3.00 [3.00–4.00]	3.00 [3.00–4.00]	0.858	−0.034 ± 0.192	[−0.418, 0.349]
	POD5		3.00 [3.00–4.00]	4.00 [3.00–4.00]	0.058	0.414 ± 0.214	[−0.014, 0.842]
	*PONV*	*n* (%)	3 (10.3)	2 (6.9)	0.640		
	*Adverse events*	*n* (%)	8 (27.59)	3 (10.34)	0.179		
	Pleural effusion		3	2			
	Pneumonia		1	0			
	Anastomotic fistula		1	0			
	Poor intestinal peristalsis		1	0			
	Delirium		1	0			
	Transfusion		1	1			
	First out-of-bed activity	days	3.21 ± 0.98	3.54 ± 1.26	0.275	−0.329 ± 0.298	[−0.927, 0.269]
	Removal of chest drainage tube	days	9.55 ± 1.96	9.54 ± 2.19	0.977	0.016 ± 0.549	[−1.084, 1.116]
	First intestinal aerofluxus	days	5.14 ± 3.79	4.61 ± 1.52	0.493	0.531 ± 0.77	[−1.012, 2.073]
	Time for discharge	days	23.69 ± 7.59	22.34 ± 3.67	0.394	1.345 ± 1.566	[−1.792, 4.481]
	Time for postoperative hospital stay	days	14.48 ± 7.06	12.38 ± 2.50	0.136	2.103 ± 1.391	[−0.684, 4.891]
	Hospital cost	CNY	50,878.03 ± 12061.19	48,808.29 ± 9510.67	0.471	2069.75 ± 2852.254	[−3644.001, 7783.5]

Data are shown as absolute numbers (*n*), percentages of the total population (*N*), and the mean ± standard error or median (interquartile range), as appropriate. POD: postoperative day; PACU: postanesthesia care unit; VAS: visual analog scale; PCIA: patient-controlled i.v. analgesia; PONV: postoperative nausea and vomiting; CNY: China Yuan.

**Table 3:** Long-term recovery data.

		Saline (*n* = 28)	Dexmedetomidine (*n* = 28)	*p* value	Difference between mean/median	95% confidence interval
SF-8	Baseline	13.10 ± 3.65	12.07 ± 2.88	0.236	1.034 ± 0.863	[−0.694, 2.763]
	1 month after surgery	27.10 ± 5.90	27.72 ± 3.50	0.628	−0.621 ± 1.274	[−3.173, 1.932]
	3 months after surgery	22.90 ± 6.17	24.62 ± 5.52	0.267	−1.724 ± 1.537	[−4.803, 1.354]

Data are reported as the mean ± standard error as appropriate. SF: short-form.

**Table 4:** Laboratory data.

			Saline (*n* = 29)	Dexmedetomidine (*n* = 29)	*p* value	Difference between mean/median	95% confidence interval
Blood cell count	*Red blood cell*	10^12^/L					
	Baseline		4.37 ± 0.50	4.38 ± 0.52	0.941	0.01 ± 0.134	[−0.258, 0.27]
	POD2		3.70 ± 0.57	3.71 ± 0.54	0.946	0.01 ± 0.146	[−0.282, 0.302]
	POD5		3.56 ± 0.57	3.58 ± 0.62	0.900	0.02 ± 0.156	[−0.293, 0.333]
	*Hemoglobin*	g/L					
	Baseline		131.59 ± 11.56	130.21 ± 16.10	0.709	−1.380 ± 3.681	[−8.753, 5.993]
	POD2		111.61 ± 14.62	109.12 ± 14.54	0.518	−2.490 ± 3.829	[−10.16, 5.18]
	POD5		106.21 ± 15.09	105.28 ± 16.97	0.826	−0.93 ± 4.217	[−9.377, 7.517]
	*White blood cell*	10^9^/L					
	Baseline		5.69 ± 1.62	5.64 ± 1.51	0.911	−0.05 ± 0.411	[−8.378, 0.774]
	POD2		11.03 ± 3.42	10.10 ± 3.44	0.314	−0.93 ± 0.901	[−2.734, 0.875]
	POD5		7.63 ± 2.61	7.86 ± 3.67	0.792	0.23 ± 0.836	[−1.445, 1.905]
	*Basophils*	10^9^/L					
	Baseline		0.21 ± 0.93	0.05 ± 0.13	0.363	−0.16 ± 0.174	[−0.509, 0.189]
	POD2		0.01 ± 0.007	0.008 ± 0.006	0.281	−0.002 ± 0.002	[−0.007, 0.002]
	POD5		0.03 ± 0.56	0.04 ± 0.12	0.925	0.01 ± 0.106	[−0.203, 0.223]
	*Eosinophil*	10^9^/L					
	Baseline		0.85 ± 2.13	0.16 ± 0.12	0.090	−0.690 ± 0.396	[−1.484, 0.396]
	POD2		0.34 ± 1.32	0.30 ± 1.31	0.908	−0.04 ± 0.345	[−0.732, 0.652]
	POD5		0.21 ± 0.16	0.17 ± 0.13	0.301	−0.04 ± 0.038	[−0.117, 0.037]
	*Monocyte*	10^9^/L					
	Baseline		0.42 ± 0.19	0.37 ± 0.16	0.283	−0.05 ± 0.046	[−0.142, 0.042]
	POD2		0.57 ± 0.23	0.56 ± 0.24	0.872	−0.01 ± 0.062	[−0.134, 0.114]
	POD5		0.57 ± 0.27	0.55 ± 0.28	0.783	−0.02 ± 0.072	[−0.165, 0.125]
	*Platelet*	10^9^/L					
	Baseline		212.83 ± 68.14	196.72 ± 64.80	0.360	−16.11 ± 17.46	[−51.09, 18.87]
	POD2		143.79 ± 40.62	153.64 ± 71.22	0.527	9.85 ± 15.23	[−20.65, 40.35]
	POD5		199.59 ± 62.86	192.48 ± 73.08	0.639	−7.11 ± 17.9	[−42.97, 28.75]
Cytokines	*CRP*	*μ*g/ml					
	Baseline		2.29 ± 1.27	2.23 ± 1.71	0.88	−0.06 ± 0.396	[−0.852, 0.7324]
	End of surgery		1.47 ± 1.31	1.57 ± 1.63	0.80	0.1 ± 0.388	[−0.678, 0.878]
	POD2		5.03 ± 0.59	5.21 ± 1.26	0.489	0.18 ± 0.258	[−0.338, 0.7]
	POD5		4.80 ± 1.81	5.08 ± 1.51	0.525	0.28 ± 0.438	[−0.6, 1.157]
	*IL-6*	pg/ml					
	Baseline		13.74 ± 8.63	11.19 ± 3.22	0.628	−2.55 ± 1.71	[−5.976, 0.877]
	End of surgery		14.88 ± 6.36	17.27 ± 7.23	0.708	2.390 ± 1.788	[−1.192, 5.972]
	POD2		15.91 ± 10.41	19.08 ± 8.11	0.59	3.17 ± .45	[−1.739, 8.079]
	POD5		9.33 ± 4.18	14.22 ± 8.64	0.140	2.890 ± 1.782	[−0.68, 6.46]
	*IL-10*	pg/ml					
	Baseline		15.79 ± 8.74	12.76 ± 7.88	0.674	−3.03 ± 2.185	[−7.408, 1.348]
	End of surgery		23.70 ± 10.71	19.92 ± 11.02	0.191	−3.78 ± 2.854	[−9.496 to 1.936]
	POD2		14.51 ± 6.94	19.39 ± 8.46	0.393	4.88 ± 2.032	[−10.95, 1.182]
	POD5		14.92 ± 10.81	12.85 ± 8.12	0.928	−2.07 ± 2.511	[−3.998, 8.137]

Data are reported as the mean ± standard error as appropriate. POD: postoperative day.

## Data Availability

The data used to support the findings of this study are available from the corresponding author upon request.
